# Herbal Polyphenolic Mixtures as Antioxidant and Cytoprotective Agents in Respiratory Diseases: Molecular Mechanisms and Therapeutic Perspectives

**DOI:** 10.3390/ijms27125298

**Published:** 2026-06-11

**Authors:** Shynggys Sergazy, Zarina Shulgau, Madiyar Nurgaziyev, Ayaulym Nurgaziyeva, Madina Baurzhan, Sayagul Kairgeldina, Alexander Gulyayev

**Affiliations:** Research Institute of Balneology and Medical Rehabilitation, Astana 010000, Kazakhstan; zarina4006@mail.ru (Z.S.); madiyar.nurgaziyev@nu.edu.kz (M.N.); anurgozhina@nu.edu.kz (A.N.); madina_baurzhan@mail.ru (M.B.); sanborocoe@mail.ru (S.K.)

**Keywords:** oxidative stress, polyphenols, herbal mixtures, respiratory diseases, cytoprotection, inflammation, Nrf2, NF-κB, pulmonary fibrosis, COPD, asthma

## Abstract

Oxidative stress is a central pathogenic mechanism in acute and chronic respiratory diseases, including asthma, chronic obstructive pulmonary disease (COPD), acute lung injury (ALI), acute respiratory distress syndrome (ARDS), and pulmonary fibrosis. Excessive production of reactive oxygen and nitrogen species (ROS/RNS), combined with impaired antioxidant defenses, contributes to epithelial and endothelial injury, inflammation, mitochondrial dysfunction, airway remodeling, and progressive loss of lung function. Plant-derived polyphenols and polyphenol-rich herbal mixtures have emerged as promising candidates for respiratory protection due to their multimodal activity. They exert effects through direct antioxidant action, enhancement of glutathione-dependent and enzymatic defenses, activation of the Nrf2/HO-1 pathway, and suppression of NF-κB, MAPK, inflammasome, and profibrotic signaling. Experimental studies have demonstrated protective effects of compounds such as quercetin, resveratrol, rosmarinic acid, epigallocatechin gallate, and phenolic-rich extracts. However, clinical translation remains limited by poor bioavailability, variability of botanical preparations, lack of standardization, and insufficient high-quality human studies. This review summarizes key mechanisms of oxidative lung injury and critically evaluates the therapeutic potential and translational challenges of herbal polyphenolic mixtures in respiratory diseases.

## 1. Introduction

Respiratory diseases remain a major cause of morbidity, impaired quality of life, and premature mortality worldwide. Although these disorders differ in etiology, clinical manifestations, and progression, many of them share several core pathogenic mechanisms, including oxidative stress, chronic or dysregulated inflammation, epithelial and endothelial injury, mitochondrial dysfunction, and abnormal tissue remodeling [1]. These overlapping processes are observed across asthma, chronic obstructive pulmonary disease (COPD), acute lung injury (ALI), acute respiratory distress syndrome (ARDS), pulmonary hypertension, and interstitial fibrotic lung disorders, suggesting that therapeutic strategies targeting common molecular pathways may have broad relevance in respiratory medicine.

Among these shared mechanisms, oxidative stress is one of the most important drivers of lung injury. The respiratory system is continuously exposed to high oxygen pressure and to numerous exogenous oxidant-generating factors, including environmental pollutants, cigarette smoke, allergens, pathogens, and inflammatory mediators. Under physiological conditions, reactive oxygen species (ROS) and reactive nitrogen species (RNS) are tightly controlled by endogenous antioxidant systems such as superoxide dismutase, catalase, glutathione peroxidase, glutathione, and redox-sensitive transcriptional regulators including nuclear factor erythroid 2-related factor 2 (Nrf2) [1,2]. When oxidant generation exceeds antioxidant capacity, oxidative injury amplifies inflammatory signaling, impairs barrier integrity, promotes apoptosis and other forms of regulated cell death, and contributes to disease progression.

This oxidative-inflammatory interplay is particularly important in chronic airway diseases. In COPD, persistent exposure to cigarette smoke and other noxious agents promotes oxidative burden, corticosteroid insensitivity, mucus hypersecretion, and progressive structural damage of the airways and lung parenchyma [3,4]. In asthma, oxidative stress contributes to epithelial activation, airway hyperresponsiveness, inflammatory cell recruitment, and remodeling, particularly in severe or steroid-resistant phenotypes [4]. Similar mechanisms are also involved in acute inflammatory lung injury, where excessive oxidant production and inflammatory amplification rapidly compromise the alveolar-capillary barrier and worsen gas exchange [1].

Because of this, antioxidant-based approaches have long been considered attractive in respiratory therapy. However, the clinical performance of conventional antioxidant strategies has often been inconsistent. One major reason is that respiratory diseases are not driven by a single damaging molecule or pathway, but by complex and interconnected networks involving oxidant stress, inflammatory transcription, innate immune activation, mitochondrial dysfunction, endothelial injury, and fibrotic signaling [3,5]. This complexity has increased interest in multitarget natural compounds that may modulate several pathogenic processes simultaneously rather than acting only as simple chemical scavengers.

Polyphenols are a large and chemically diverse class of plant-derived metabolites that include flavonoids, phenolic acids, stilbenes, lignans, and tannins. Over the last two decades, they have attracted considerable attention because of their antioxidant, anti-inflammatory, anti-apoptotic, and anti-fibrotic properties. Importantly, their biological activity extends well beyond direct ROS neutralization. Many polyphenols regulate intracellular signaling pathways involved in cytoprotection, including activation of Nrf2 and inhibition of NF-κB, MAPK, and inflammasome-associated cascades [6,7]. As a result, these compounds may reinforce endogenous antioxidant defenses while simultaneously suppressing inflammatory and profibrotic responses in lung tissue.

A growing body of experimental evidence supports this concept. Preclinical studies have shown that individual polyphenols and polyphenol-rich plant extracts can reduce oxidative injury, suppress inflammatory mediator production, preserve epithelial and endothelial function, and attenuate pathological remodeling in several respiratory conditions. For example, tea polyphenols have shown protective effects in sepsis-associated lung injury through mitochondria-related mechanisms, while broader recent syntheses indicate beneficial activity of polyphenols in asthma, COPD, pulmonary fibrosis, pulmonary hypertension, and related disorders [7,8,9].

At the same time, interest is increasingly shifting from isolated compounds toward herbal polyphenolic mixtures, which may provide additive or synergistic effects through multi-component, pleiotropic interactions [7,9]. This shift is scientifically important for at least two reasons. First, medicinal plants are rarely used clinically as pure single-molecule interventions; they are more commonly administered as extracts, fractions, or formulated combinations containing multiple phenolic constituents. Second, respiratory diseases themselves are biologically heterogeneous, and it is plausible that mixtures with complementary antioxidant, anti-inflammatory, and barrier-protective effects may be better suited to this complexity than reductionist one-target approaches.

Nevertheless, this therapeutic promise must be interpreted critically. Botanical heterogeneity, differences in extraction procedures, uncertain pharmacokinetics, limited standardization, and variable bioavailability remain substantial barriers to clinical translation [7,10]. Therefore, a rigorous evaluation of both mechanistic and experimental evidence is essential before herbal polyphenolic mixtures can be considered credible candidates for respiratory therapy.

In this context, the present review examines the role of herbal polyphenolic mixtures as antioxidant and cytoprotective agents in respiratory diseases. We first summarize the pathophysiology of oxidative and cytotoxic lung injury, with particular emphasis on ROS/RNS generation, endogenous antioxidant imbalance, and redox-sensitive inflammatory pathways. We then discuss the principal classes of polyphenols relevant to respiratory protection, followed by representative compounds and multi-component herbal formulations. Finally, we critically evaluate the available experimental and clinical evidence, identify major translational limitations, and outline future directions for the development of scientifically grounded phytotherapeutic strategies in respiratory medicine.

Importantly, this work is presented as a narrative review based on a structured literature search and critical evaluation of published data. While several recent reviews have addressed the biological activity of individual polyphenols in respiratory diseases, a comprehensive synthesis specifically focused on herbal polyphenolic mixtures as integrated multi-component systems remains limited. In addition, previous literature often discusses oxidative stress, inflammation, mitochondrial dysfunction, and tissue remodeling separately, without fully integrating these processes into a unified mechanistic and translational framework. The present review addresses this gap by emphasizing herbal polyphenolic mixtures as pleiotropic therapeutic systems and by integrating pathway-level and organelle-level mechanisms—including mitochondrial dysfunction, inflammasome activation, and epithelial–endothelial barrier injury—within a single critical perspective.

## 2. Pathophysiology of Oxidative and Cytotoxic Damage in Respiratory Diseases

### 2.1. Sources of Reactive Oxygen Species (ROS) and Reactive Nitrogen Species (RNS) in the Lung

The lung is especially vulnerable to oxidative stress because it is continuously exposed to high oxygen concentrations and to a broad range of exogenous oxidant-generating agents, including cigarette smoke, air pollutants, allergens, nanoparticles, and respiratory pathogens. At the same time, the inflamed respiratory microenvironment contains multiple endogenous sources of ROS and RNS generated by structural and immune cells. As a result, oxidative stress in respiratory disease is not a single event but a complex and self-amplifying process involving epithelial cells, endothelial cells, alveolar macrophages, neutrophils, eosinophils, fibroblasts, and vascular smooth muscle cells [1,2].

Among endogenous ROS sources, mitochondria are particularly important. Under physiological conditions, mitochondrial electron transport generates low levels of superoxide that are neutralized by antioxidant systems. In diseased lungs, however, mitochondrial dysfunction leads to excessive electron leakage, increased ROS formation, loss of membrane potential, impaired ATP production, and activation of cell death and inflammatory pathways. These events are highly relevant in acute lung injury, sepsis-associated lung damage, COPD, and fibrotic remodeling, where mitochondrial stress contributes to barrier disruption and tissue injury [8,11].

Another major source of ROS in the respiratory system is the nicotinamide adenine dinucleotide phosphate (NADPH) oxidase (NOX) family. Unlike mitochondria, whose primary function is ATP production, NOX enzymes are dedicated ROS-generating systems. NOX2 is highly expressed in phagocytes and mediates the respiratory burst during host defense, whereas NOX4 is constitutively active in epithelial cells, endothelial cells, and fibroblasts and is strongly implicated in chronic tissue remodeling and fibrosis [12,13]. Activation of NOX enzymes by cytokines, pathogens, cigarette smoke, and mechanical stress promotes superoxide and hydrogen peroxide production, leading to epithelial injury, endothelial dysfunction, leukocyte recruitment, and activation of NF-κB, MAPK, and transforming growth factor-β (TGF-β) signaling pathways.

Several additional enzymatic systems contribute to oxidative stress in the lung. Xanthine oxidase catalyzes purine metabolism and produces superoxide and hydrogen peroxide, particularly during ischemia–reperfusion and inflammatory injury [14]. Myeloperoxidase released from activated neutrophils converts hydrogen peroxide and chloride ions into hypochlorous acid, a highly reactive oxidant that damages proteins, lipids, and extracellular matrix components [15]. Endothelial nitric oxide synthase (eNOS) may become uncoupled under conditions of tetrahydrobiopterin deficiency, generating superoxide instead of nitric oxide and further amplifying vascular oxidative stress [16].

Reactive nitrogen species are equally important mediators of pulmonary injury. Nitric oxide (NO) plays essential roles in bronchodilation, vascular regulation, and host defense under normal conditions [17]. However, during inflammation, inducible nitric oxide synthase (iNOS) is markedly upregulated in epithelial cells, macrophages, and neutrophils, resulting in sustained NO production. When NO reacts with superoxide, it forms peroxynitrite, a potent oxidant capable of inducing lipid peroxidation, protein tyrosine nitration, DNA strand breaks, and mitochondrial dysfunction [18]. Increased nitrotyrosine formation has been demonstrated in airway tissues of patients with asthma and COPD, linking nitrosative stress to corticosteroid resistance, epithelial dysfunction, and persistent inflammation [4].

### 2.2. Imbalance of Antioxidant Defenses

Under normal conditions, the lung is protected by a well-developed antioxidant network that includes enzymatic defenses such as superoxide dismutase (SOD), catalase (CAT), glutathione peroxidase (GPx), heme oxygenase-1 (HO-1), and peroxiredoxins, as well as non-enzymatic molecules such as glutathione (GSH), uric acid, thioredoxin, and dietary antioxidants. Together, these systems maintain redox homeostasis by neutralizing superoxide, hydrogen peroxide, lipid peroxides, and electrophilic intermediates before they cause structural or signaling damage [2,19].

In respiratory disease, this protective balance is frequently disturbed. Chronic oxidant exposure and persistent inflammation can consume antioxidant reserves, suppress antioxidant enzyme expression, impair glutathione recycling, and reduce the capacity of the respiratory epithelium to respond to oxidative challenge. This imbalance has been reported in COPD, asthma, acute inflammatory lung injury, and fibrotic lung disorders, where reduced antioxidant tone is associated with more severe inflammation, greater tissue injury, and impaired resolution of damage [3,20].

Glutathione is one of the most important intracellular antioxidant molecules in the lung. It directly scavenges reactive species, participates in detoxification reactions, preserves thiol redox balance, and supports the activity of glutathione peroxidases and related enzymes. Depletion of glutathione has been shown to increase susceptibility of epithelial and endothelial cells to oxidant-induced apoptosis and inflammatory signaling [20,21]. Reduced activity of superoxide dismutase, catalase, and glutathione peroxidase limits the conversion of superoxide and hydrogen peroxide into less reactive products, thereby promoting lipid peroxidation, mitochondrial dysfunction, and amplification of redox-sensitive inflammatory pathways [20].

The regulation of antioxidant defenses is closely linked to the transcription factor nuclear factor erythroid 2-related factor 2 (Nrf2), which controls the expression of genes involved in glutathione synthesis, phase II detoxification, antioxidant enzyme induction, and cytoprotective adaptation. Under basal conditions, Nrf2 is retained in the cytoplasm by Kelch-like ECH-associated protein 1 (Keap1) and targeted for proteasomal degradation. Oxidative or electrophilic stress disrupts this interaction, allowing Nrf2 to translocate to the nucleus and activate antioxidant response element-dependent genes, including heme oxygenase-1, NAD(P)H quinone oxidoreductase 1, glutamate-cysteine ligase, and glutathione S-transferases. Impaired Nrf2 signaling has been associated with reduced antioxidant capacity, persistent inflammation, and increased susceptibility to oxidative injury in COPD, acute lung injury, and pulmonary fibrosis [22,23].

Because oxidative injury in respiratory disease reflects both increased oxidant production and weakened antioxidant defenses, restoration of the endogenous redox network represents a major therapeutic objective. This concept has driven growing interest in polyphenols, many of which act not only as direct radical scavengers but also as modulators of endogenous defense systems. Experimental studies have shown that polyphenols can activate Nrf2-dependent transcription, enhance glutathione synthesis, and increase the expression and activity of antioxidant enzymes such as superoxide dismutase, catalase, glutathione peroxidase, and heme oxygenase-1 [8,24]. These mechanisms provide a strong molecular rationale for the use of polyphenol-rich herbal mixtures as pleiotropic cytoprotective agents in respiratory diseases.

### 2.3. Molecular Pathways Linking Oxidative Stress to Inflammation

Oxidative stress is not only a consequence of respiratory inflammation but also an active signaling driver that links oxidant injury to cytokine production, immune cell recruitment, mucus hypersecretion, and tissue remodeling. This connection is mediated through several redox-sensitive pathways, among which NF-κB, Nrf2, and MAPK are the most extensively studied in respiratory disease [2].

Among redox-sensitive signaling pathways, nuclear factor-κB (NF-κB) is one of the most extensively studied mediators linking oxidative stress to inflammation. Under resting conditions, NF-κB dimers are retained in the cytoplasm by inhibitor of κB (IκB) proteins. Oxidative stress and pro-inflammatory stimuli activate the IκB kinase complex, resulting in phosphorylation and proteasomal degradation of IκB and subsequent nuclear translocation of NF-κB [25]. Activated NF-κB induces transcription of numerous inflammatory mediators, including tumor necrosis factor-α, interleukin-1β, interleukin-6, inducible nitric oxide synthase, cyclooxygenase-2, and adhesion molecules. Persistent NF-κB activation has been documented in airway epithelial cells and alveolar macrophages from patients with asthma and COPD and is closely associated with chronic inflammation, mucus hypersecretion, and tissue injury [26].

Mitogen-activated protein kinase (MAPK) cascades, including extracellular signal-regulated kinase (ERK), c-Jun N-terminal kinase (JNK), and p38 MAPK, are also highly responsive to oxidative stress. ROS modulate these pathways through reversible oxidation of upstream kinases and phosphatases, resulting in sustained phosphorylation and activation of downstream transcription factors such as activator protein-1 (AP-1) [27]. Activation of p38 MAPK and JNK promotes cytokine production, epithelial apoptosis, and remodeling, whereas ERK signaling contributes to cell proliferation, goblet cell hyperplasia, and fibroblast activation. Experimental studies have shown that pharmacological suppression of MAPK signaling reduces inflammatory cytokine release and attenuates lung injury in asthma, acute lung injury, and pulmonary fibrosis models [27,28,29,30].

The NLR family pyrin domain-containing 3 (NLRP3) inflammasome is a multiprotein signaling complex that links oxidative stress to innate immune activation. It consists of the sensor protein NLRP3, the adaptor apoptosis-associated speck-like protein containing a CARD (ASC), and pro-caspase-1. ROS, mitochondrial dysfunction, oxidized mitochondrial DNA, and ion flux disturbances promote inflammasome assembly, leading to caspase-1 activation and maturation of interleukin-1β and interleukin-18 [31,32]. Excessive NLRP3 activation contributes to neutrophilic inflammation, epithelial damage, and fibrotic remodeling in COPD, asthma, acute lung injury, and idiopathic pulmonary fibrosis [33]. Because many polyphenols suppress mitochondrial ROS and inflammasome activation, this pathway represents an important mechanistic target of phytochemical intervention [34,35,36,37].

Oxidative stress also contributes to corticosteroid resistance, particularly in severe asthma and COPD. Reactive aldehydes and peroxynitrite reduce the activity and expression of histone deacetylase 2 (HDAC2), a key co-repressor required for glucocorticoid-mediated suppression of inflammatory gene transcription [38,39]. Loss of HDAC2 impairs glucocorticoid responsiveness and allows persistent NF-κB-driven inflammation. This mechanism has important therapeutic implications because compounds that restore redox balance and preserve HDAC2 function may improve steroid sensitivity [38,40]. Polyphenols capable of suppressing ROS-dependent signaling may therefore exert both direct anti-inflammatory effects and indirect steroid-sensitizing actions, as shown experimentally for curcumin, which restored corticosteroid inhibition of cytokine release in cigarette-smoke-exposed monocytes/macrophages [41].

### 2.4. Cytoprotective Targets in the Respiratory Epithelium and Endothelium

The respiratory epithelium and pulmonary endothelium are highly specialized and metabolically active interfaces that regulate gas exchange, innate immune surveillance, fluid homeostasis, coagulation, leukocyte trafficking, and tissue repair. Rather than serving as passive barriers, these cell layers continuously sense and respond to inhaled and circulating stress signals. Because they are directly exposed to oxidants, inflammatory mediators, pathogens, and toxic metabolites, epithelial and endothelial cells are among the earliest and most important targets of injury in both acute and chronic respiratory diseases [42,43]. Cytoprotection in the lung therefore depends not only on reducing oxidative burden, but also on preserving barrier integrity, mitochondrial function, adaptive stress signaling, and the capacity for controlled repair [11,43].

One of the most important cytoprotective targets is the Nrf2/HO-1 pathway. In epithelial and endothelial cells, activation of Nrf2 induces a coordinated transcriptional response that enhances glutathione synthesis, phase II detoxification, antioxidant enzyme expression, and resistance to oxidative injury. Heme oxygenase-1 (HO-1), a major downstream effector of Nrf2, degrades heme to biliverdin, carbon monoxide, and ferrous iron and exerts anti-inflammatory, antioxidant, and anti-apoptotic effects [22,23].

A second major cytoprotective target is mitochondrial homeostasis. Epithelial and endothelial cells rely on intact mitochondrial function to maintain ATP production, redox balance, calcium handling, and survival signaling. Excess mitochondrial ROS, membrane depolarization, impaired mitophagy, and release of pro-apoptotic factors such as cytochrome c lead to barrier disruption, inflammatory amplification, and defective tissue repair [11]. In a sepsis-induced lung injury model, tea polyphenols promoted translocation of DJ-1 to mitochondria, reduced mitochondrial ROS production, preserved membrane potential, and restored antioxidant enzyme activity, resulting in significant attenuation of pulmonary edema and inflammatory injury [8]. These findings indicate that polyphenol-mediated cytoprotection may involve preservation of mitochondrial integrity and intracellular stress adaptation mechanisms rather than solely direct radical-scavenging activity.

Another critical cytoprotective target is the epithelial and endothelial barrier itself. Epithelial integrity depends on tight junction proteins such as zonula occludens-1 (ZO-1), occludin, and claudins, whereas endothelial integrity is maintained primarily by adherens junction proteins including vascular endothelial cadherin (VE-cadherin). Oxidative stress disrupts these structures through lipid peroxidation, cytoskeletal contraction, and activation of inflammatory signaling pathways, resulting in increased permeability, protein-rich edema, and enhanced leukocyte infiltration [42,43,44,45,46]. In ALI and ARDS, barrier failure is a central event leading to diffuse alveolar damage and severe impairment of gas exchange, while in chronic airway disorders persistent epithelial fragility contributes to abnormal repair and remodeling [47].

Inflammasome regulation is also increasingly recognized as a key cytoprotective mechanism. Activation of the NLRP3 inflammasome in epithelial and endothelial cells leads to caspase-1 activation, maturation of interleukin-1β and interleukin-18, and pyroptotic cell death, thereby amplifying oxidative and inflammatory injury [31,32]. This pathway is particularly relevant in cigarette smoke-induced COPD and acute inflammatory lung injury. Fu et al. emphasized that polyphenols and flavonoids can suppress inflammasome signaling, thereby preserving epithelial and endothelial function under chronic oxidant stress [48].

Stress-activated kinase pathways, particularly p38 MAPK, also represent important cytoprotective targets. These pathways regulate cytokine production, apoptosis, mucus hypersecretion, and oxidative-inflammatory amplification. In a cigarette smoke-induced acute lung injury model, apple polyphenols significantly reduced pulmonary edema, inflammatory cell infiltration, malondialdehyde accumulation, and p38 MAPK activation, while restoring superoxide dismutase activity and glutathione levels [49]. These results demonstrate that cytoprotection may be achieved through simultaneous suppression of redox-sensitive inflammatory signaling and reinforcement of endogenous antioxidant defenses.

Finally, cytoprotection in the respiratory epithelium and endothelium must be considered in the context of apoptosis, senescence, and abnormal tissue remodeling. Persistent oxidative stress induces DNA damage, mitochondrial dysfunction, and activation of p53- and caspase-dependent pathways, leading to epithelial cell loss and impaired regeneration [50]. In fibroblasts, oxidant-sensitive transforming growth factor-β signaling promotes myofibroblast differentiation, α-smooth muscle actin expression, and collagen deposition [13]. Senescent epithelial and endothelial cells acquire a pro-inflammatory secretory phenotype that sustains cytokine release and extracellular matrix remodeling [51]. Accordingly, interventions that prevent apoptosis, preserve barrier function, and attenuate profibrotic signaling may provide long-term benefits beyond the reduction in acute inflammatory injury.

In summary, the most relevant cytoprotective targets in respiratory epithelial and endothelial cells include the Nrf2/HO-1 antioxidant system, mitochondrial quality control, epithelial–endothelial barrier maintenance, inflammasome regulation, redox-sensitive kinase pathways, and anti-remodeling mechanisms. These interconnected targets provide the mechanistic basis for understanding how polyphenols and polyphenol-rich herbal mixtures may protect lung tissues in both acute and chronic respiratory disorders. Collectively, the key molecular pathways involved in oxidative stress and cytoprotection in respiratory diseases are summarized in Table 1.

## 3. Polyphenols as Multi-Target Cytoprotective Agents in Respiratory Diseases

### 3.1. Classification of Polyphenols Relevant to Respiratory Protection

Polyphenols are a large and structurally diverse group of plant secondary metabolites characterized by one or more phenolic rings with varying patterns of hydroxylation, methylation, glycosylation, and polymerization [58]. Their pharmacological relevance in respiratory disease extends far beyond direct free radical scavenging. Experimental studies have shown that polyphenols modulate multiple interconnected pathways involved in oxidative stress, inflammation, mitochondrial dysfunction, and fibrotic remodeling, including activation of Nrf2-dependent antioxidant responses, suppression of NF-κB and MAPK signaling, inhibition of NLRP3 inflammasome activation, and attenuation of transforming growth factor-β (TGF-β)-driven profibrotic signaling [34,35,36,44]. Such broad mechanistic activity is particularly relevant in respiratory diseases, where epithelial injury, endothelial dysfunction, inflammatory amplification, and abnormal tissue remodeling occur simultaneously.

The main polyphenol classes relevant to respiratory protection include flavonoids, phenolic acids, stilbenes, lignans, and tannins. Among these, flavonoids represent the most extensively studied group in respiratory models and include flavonols such as quercetin, flavanones such as hesperidin, flavan-3-ols such as catechins and epigallocatechin-3-gallate (EGCG), and more complex glycosylated derivatives. Phenolic acids, including rosmarinic acid and caffeic acid derivatives, are also highly relevant because of their combined antioxidant and anti-inflammatory properties. Stilbenes are represented mainly by resveratrol, which has been widely investigated for its anti-inflammatory and anti-fibrotic effects. Tannins and polymeric phenolics are especially important when the focus shifts from isolated molecules to botanical extracts and herbal mixtures, since many plant preparations contain complex tannin-rich fractions that may influence several pathways at once [10,37,59,60,61,62,63].

From a pharmacological perspective, classification based on dominant biological mechanisms is often more informative than classification based solely on chemical scaffold. In experimental respiratory systems, polyphenols have been shown to activate endogenous antioxidant responses, suppress NF-κB- and stress-kinase–driven inflammatory signaling, limit inflammasome-associated injury, preserve mitochondrial homeostasis, and attenuate profibrotic remodeling; however, the strength of evidence for each pathway varies by compound and by disease model [8,64,65,66]. In practice, most polyphenols studied in respiratory models display overlapping rather than exclusive mechanisms, which is one reason they remain attractive candidates in asthma, COPD, acute lung injury, and pulmonary fibrosis, where disease progression is driven by interconnected oxidative, inflammatory, and remodeling pathways.

An important translational consideration is that medicinal plants and botanical preparations rarely deliver a single polyphenol in isolation. Standardized extracts usually contain multiple flavonoids, phenolic acids, tannins, and non-phenolic co-metabolites, so their biological activity may reflect additive, synergistic, or occasionally antagonistic interactions that cannot be predicted from any one constituent alone [67]. Accordingly, in the present review, polyphenols are considered at two complementary levels: first, as individual molecules with relatively well-defined mechanisms, and second, as components of polyphenol-rich preparations whose translational value depends on chemical characterization, standardization, and formulation quality.

### 3.2. Major Individual Polyphenols with Respiratory Cytoprotective Potential

#### 3.2.1. Quercetin

Quercetin is one of the most extensively studied flavonols in respiratory research and is abundant in onions, apples, berries, and numerous medicinal plants, and it has been extensively investigated in experimental models of inflammatory and infectious lung injury [68]. In lipopolysaccharide-induced acute lung injury, quercetin reduced pulmonary inflammation and tissue injury by suppressing SIRT1-mediated PKM2 nuclear accumulation, which was associated with lower inflammatory activation in lung tissue [55]. Quercetin also attenuated methylglyoxal-induced inflammatory injury through activation of the AMPK/SIRT1 pathway and inhibition of NF-κB signaling, supporting its role as a regulator of oxidative and inflammatory stress responses [69]. In infectious respiratory models, quercetin protected mice against *Streptococcus pneumoniae* infection by inhibiting pneumolysin activity, reducing bacterial virulence-associated lung injury, and improving survival outcomes [70]. In addition to its anti-inflammatory and anti-infective effects, quercetin is relevant to fibrotic remodeling because TGF-β1-driven fibroblast-to-myofibroblast differentiation is a key mechanism of extracellular matrix deposition and pulmonary fibrosis; experimentally, TGF-β1-stimulated myofibroblast differentiation has been linked to hyaluronan-facilitated EGFR and CD44 co-localization in lipid rafts [71]. Taken together, available evidence suggests that quercetin exerts broad respiratory cytoprotective activity through modulation of inflammatory, oxidative, and fibroproliferative pathways.

#### 3.2.2. Resveratrol

Resveratrol is a natural stilbene polyphenol found in grapes, berries, and several medicinal plants, and it has demonstrated significant anti-inflammatory, antioxidant, and anti-fibrotic effects in experimental models of interstitial and inflammatory lung diseases. In bleomycin-induced pulmonary fibrosis models, resveratrol attenuated epithelial–mesenchymal transition (EMT), collagen deposition, and pulmonary remodeling through suppression of the TGF-β1/Smad3 and TLR4/NF-κB signaling pathways [72]. Similarly, resveratrol reduced pulmonary fibrosis and inflammatory responses by suppressing HIF-1α and NF-κB expression in bleomycin-treated animals, further supporting its role in regulation of pro-fibrotic and inflammatory signaling cascades [73]. Earlier mechanistic studies demonstrated that resveratrol inhibited transforming growth factor-β-induced differentiation of human lung fibroblasts into myofibroblasts through inhibition of ERK/Akt signaling and restoration of PTEN activity, indicating direct effects on fibroblast activation and extracellular matrix remodeling [74]. In inflammation-associated lung injury models, resveratrol suppressed NLRP3 inflammasome activation and attenuated particulate matter-induced pulmonary inflammation and fibrosis in mice [75]. Additionally, resveratrol modulated miR-21/MAPK/AP-1 signaling and reduced fibrogenic activation in pulmonary fibrosis models, highlighting its regulatory role at both transcriptional and post-transcriptional levels [76]. Overall, experimental evidence supports resveratrol as a pleiotropic modulator of inflammatory and profibrotic signaling in lung disease models.

#### 3.2.3. Rosmarinic Acid

Rosmarinic acid is a phenolic acid derivative abundant in rosemary (*Salvia rosmarinus*), perilla, basil, lemon balm, and other Lamiaceae plants, and it has demonstrated substantial antioxidant, anti-inflammatory, and airway-protective activity in experimental respiratory disease models. In allergic asthma, rosmarinic acid alleviated airway inflammation through modulation of the gut–lung axis, reducing eosinophilic infiltration, mucus production, bronchoconstriction, and Th2/ILC2-mediated inflammatory responses. Mechanistically, these effects were associated with suppression of TLR4/NF-κB-mediated pulmonary inflammation and oxidative stress, together with modulation of gut microbiota-derived short-chain fatty acids and lipopolysaccharide signaling [77]. In a separate experimental model of H1N1 virus-mediated acute lung injury, rosmarinic acid markedly reduced inflammatory cytokine production, alveolar epithelial apoptosis, and pulmonary tissue injury through inhibition of NF-κB and p38 MAPK signaling pathways and activation of the h-PGDS-PGD2-HO-1 cytoprotective axis [78]. Antioxidant effects of rosmarinic acid were further demonstrated in experimental allergic asthma with oxidative lung injury, where treatment significantly reduced ROS production, downregulated NOX-2 and NOX-4 expression, and restored antioxidant enzyme activity, including superoxide dismutase, glutathione peroxidase, and catalase [54]. In paraquat-induced lung injury, rosmarinic acid attenuated inflammatory cell infiltration and pulmonary tissue damage while suppressing IL-1β, TNF-α, and TLR9 expression, supporting its protective effects against toxic inflammatory lung injury [79]. In addition to anti-inflammatory actions, rosmarinic acid has also been implicated in regulation of fibroblast survival and fibrotic remodeling. Experimental studies in human lung fibroblasts demonstrated that rosmarinic acid potentiated carnosic acid-induced apoptosis and contributed to attenuation of collagen deposition and oxidative stress in bleomycin-induced pulmonary fibrosis models [80]. These studies demonstrate that rosmarinic acid influences several interconnected pathways involved in oxidative injury, airway inflammation, and pulmonary remodeling.

#### 3.2.4. Epigallocatechin-3-Gallate (EGCG)

Epigallocatechin-3-gallate (EGCG), the major catechin polyphenol of green tea, has demonstrated broad antioxidant, anti-inflammatory, immunomodulatory, and cytoprotective activity in experimental respiratory disease models. In lipopolysaccharide-induced acute lung injury (ALI), EGCG significantly attenuated pulmonary edema, neutrophil infiltration, oxidative damage, and inflammatory cytokine production while promoting macrophage polarization toward the anti-inflammatory M2 phenotype. These effects were associated with increased Krüppel-like factor 4 (KLF4) expression, suppression of inducible nitric oxide synthase and pro-inflammatory cytokines, and enhancement of lung regenerative markers including Ki67 and angiopoietin-1 [81]. In experimental asthma, EGCG ameliorated airway hyperresponsiveness, inflammatory cell infiltration, mucus hypersecretion, oxidative stress, and epithelial apoptosis through inhibition of the TNF-α/TNF-R1/NLRP3 signaling pathway. Mechanistically, EGCG reduced reactive oxygen species production, restored mitochondrial membrane potential, and suppressed activation of NLRP3 inflammasome-associated inflammatory signaling in lung tissues and airway cells [82]. Protective effects of EGCG against inflammasome-mediated lung injury were also demonstrated in acute pancreatitis-associated pulmonary damage, where EGCG suppressed mitochondrial reactive oxygen species (mtROS) generation, reduced oxidized mitochondrial DNA accumulation, and inhibited NLRP3 inflammasome activation in alveolar macrophages [83]. In bacterial pneumonia models caused by *Pseudomonas aeruginosa*, EGCG reduced pulmonary tissue injury and improved survival by suppressing quorum-sensing-regulated virulence factors and reducing inflammatory cytokine production, including TNF-α, IL-1β, IL-6, and IL-17 [84]. Earlier studies further demonstrated that EGCG attenuated paraquat-induced acute lung injury through inhibition of TLR2/TLR4/TLR9-mediated NF-κB activation and suppression of inflammatory cytokine release in lung tissues and pulmonary epithelial cells [85]. Taken together, these studies suggest that EGCG may protect lung tissues through combined antioxidant, anti-inflammatory, mitochondrial, and immunomodulatory mechanisms.

#### 3.2.5. Curcumin

Curcumin has attracted substantial attention in respiratory pharmacology because of its combined antioxidant and anti-fibrotic activity. In idiopathic pulmonary fibrosis models, the curcumin analogue EF24 prevented bleomycin-induced alveolar epithelial cell senescence by activating PTEN and suppressing AKT/mTOR/NF-κB signaling. EF24 reduced senescence markers, including SA-β-gal, PAI-1, p21, and senescence-associated secretory phenotype factors, improved mitochondrial dysfunction through induction of mitophagy, and attenuated pulmonary fibrosis in both acute and chronic phases of experimental IPF [86]. Curcumin has also been shown to regulate extracellular matrix remodeling in pulmonary fibrosis. In bleomycin-induced lung fibrosis and TGF-β-stimulated fibroblast models, curcumin reduced collagen, fibronectin, MMP-2, MMP-9, and basement membrane protein deposition, suggesting that it can limit pathological matrix accumulation and alveolar septal thickening during fibrotic remodeling [87]. Additional fibroblast-based evidence indicates that curcumin inhibits fibrotic progression by reducing fibroblast activity and lowering TGF-β1, α-SMA, collagen I, and collagen III expression, while also improving inflammatory and oxidative stress parameters [88]. In radiation-induced lung pneumonitis and fibrosis, curcumin downregulated IL-4, IL-4 receptor-α1, DUOX1, and DUOX2 expression and mitigated histopathological pneumonitis and fibrosis, supporting its protective role against oxidant enzyme-driven chronic lung injury [89]. Recent toxic lung injury studies further show that curcumin protects against malathion-induced pulmonary damage by enhancing Nrf2/HO-1 antioxidant signaling and suppressing NF-κB/TNF-α and COX-2 inflammatory pathways, thereby reducing oxidative damage, inflammatory cytokine expression, hydroxyproline accumulation, and histopathological fibrosis [90]. Curcumin also attenuated pulmonary fibrosis by regulating the miR-29a-3p/DNMT3A axis, reducing collagen I and fibronectin expression, and modulating mitochondrial function in pulmonary fibroblasts [91]. Together, these observations position curcumin as a mechanistically versatile candidate for modulation of fibrotic and inflammatory lung injury. However, interpretation of curcumin data requires caution because poor aqueous solubility, rapid metabolism, low oral bioavailability, and formulation-dependent pharmacokinetics remain major obstacles to clinical translation.

#### 3.2.6. Hesperidin

Hesperidin is a citrus-derived flavanone glycoside that has shown antioxidant, anti-inflammatory, immunomodulatory, and anti-fibrotic activity in experimental respiratory disease models. In bleomycin-induced pulmonary fibrosis, hesperidin alleviated fibrotic remodeling by inhibiting lung fibroblast senescence through suppression of the IL-6/STAT3 signaling pathway. This effect was associated with reduced expression of senescence markers p53, p21, and p16, decreased α-smooth muscle actin expression, fewer senescence-associated β-galactosidase-positive cells, and attenuation of pulmonary fibrosis in vivo and in senescent MRC-5 fibroblasts in vitro [92]. Hesperidin has also demonstrated protective effects in acute inflammatory lung injury associated with mechanical ventilation. In experimental models of ventilator-induced lung injury, hesperidin reduced inflammatory cell recruitment into the airways, decreased CCL-2, TNF-α, IL-12, and myeloperoxidase activity, attenuated oxidative damage, and helped restore antioxidant enzyme activity [93,94]. In respiratory syncytial virus-induced bronchiolitis, hesperidin reduced lung inflammation and mucus secretion by inhibiting the Jagged1/Notch1 pathway and promoting macrophage polarization toward an M2 phenotype, as shown by decreased IL-4, IL-6, TNF-α, iNOS, Jagged1, and Notch1 expression and increased IL-10, Arg-1, and M2 macrophage proportion [95]. More recently, multi-omics analysis in hypoxia-induced high-altitude pulmonary hypertension demonstrated that hesperidin reduced mean pulmonary arterial pressure and pulmonary vascular remodeling while modulating the gut–lung axis, intestinal inflammation, choline metabolism, and pulmonary expression of EGFR, c-Fos, and WASF1 [96].

#### 3.2.7. Tannins and Polymeric Phenolics

Tannins and polymeric phenolics are highly relevant to respiratory phytotherapy because many plant-derived preparations contain proanthocyanidins, hydrolysable tannins, ellagitannin-related compounds, and other high-molecular-weight phenolic fractions. In experimental acute lung injury, grape seed proanthocyanidin attenuated LPS-induced pulmonary pathological changes, reduced inflammatory cytokine expression, decreased recruitment of monocyte-derived macrophages, and promoted macrophage polarization from a pro-inflammatory M1 phenotype toward an M2a phenotype through activation of the TREM2/PI3K/Akt pathway [97]. Procyanidin B2, another representative oligomeric flavanol, alleviated LPS-induced acute lung injury by reducing lung edema and histopathological injury, suppressing IL-1β, TNF-α, and IL-6 production, inhibiting M1 macrophage polarization, and reducing pyroptosis through downregulation of the NF-κB/JAK2/STAT1 and NLRP3/caspase-1/ASC signaling pathways [98]. In virus-associated lung injury, tannin-rich Chebulae Fructus extract and its major active constituent chebulinic acid reduced H1N1-induced pulmonary viral burden, inflammatory injury, apoptosis, and metabolic-inflammatory dysregulation by inhibiting the IDO1–kynurenine axis and suppressing RIG-I-dependent activation of NF-κB, p38/JNK/ERK MAPK, and JAK/STAT pathways [99]. Tannin-derived compounds may also influence post-infectious and remodeling processes. In an influenza-induced lung fibrosis model, pharmacological inhibition of protein disulfide isomerase A3 by punicalagin reduced lung fibrosis and improved oxygen saturation, while inhibition of the downstream osteopontin/SPP1 axis decreased virus-induced fibrotic remodeling [100]. Oligomeric proanthocyanidin further attenuated asthma severity and airway remodeling in both clinical and experimental settings by reducing epithelial-derived cytokines, intracellular ROS signaling, eotaxin-1, and TGF-β1, thereby limiting airway smooth muscle proliferation through modulation of epithelial–smooth muscle cell interactions and p38-associated signaling [101].

Taken together, these representative compounds illustrate the central principle of respiratory polyphenol pharmacology: their therapeutic value arises not from a single shared mechanism, but from convergence on an interconnected network of targets that includes Nrf2-dependent antioxidant defenses, NF-κB and MAPK signaling, mitochondrial preservation, inflammasome regulation, modulation of ferroptosis and pyroptosis, and attenuation of transforming growth factor-β-driven remodeling. A structured overview of the major individual polyphenols discussed in this section, including their chemical classification, principal molecular mechanisms, and respiratory applications, is presented in Table 2.

### 3.3. Why Herbal Polyphenolic Mixtures May Offer Advantages over Single Compounds

Although individual polyphenols are useful for defining specific molecular mechanismsp, clinically relevant botanical preparations are inherently more complex. Standardized herbal extracts often contain multiple flavonoids, phenolic acids, tannins, proanthocyanidins, and other coexisting metabolites that may interact with overlapping but non-identical molecular targets. This complexity is pharmacologically relevant in respiratory disease because oxidative stress, inflammation, epithelial and endothelial injury, mitochondrial dysfunction, immune dysregulation, and tissue remodeling are closely interconnected rather than isolated processes. Experimental and translational studies suggest that herbal preparations and polyphenol-rich mixtures can simultaneously modulate inflammatory signaling, oxidative stress responses, airway remodeling, and immune activation in respiratory disorders [102,103]. Similarly, grape seed proanthocyanidin attenuated lipopolysaccharide-induced acute lung injury by reducing inflammatory cytokine production and promoting macrophage polarization through the TREM2/PI3K/Akt pathway [97], while oligomeric proanthocyanidin reduced airway remodeling and oxidative injury in asthma by suppressing epithelial-derived cytokines, ROS-associated signaling, epithelial TGF-β1 release, and airway smooth muscle proliferation [101]. Collectively, these findings support the concept that the potential advantage of polyphenol-rich mixtures lies not in nonspecific antioxidant activity alone, but in the simultaneous modulation of several disease-relevant pathways.

At the same time, the possible advantages of polyphenol-rich mixtures should not be overstated. Additive or synergistic effects are biologically plausible, but complex extracts may also introduce variability in phytochemical composition, uncertain pharmacokinetics, batch-to-batch inconsistency, and difficulty in identifying the constituents primarily responsible for biological activity. Consequently, the scientific and translational value of herbal polyphenolic mixtures depends critically on rigorous phytochemical characterization, quantitative standardization, detailed dose reporting, and clear mechanistic linkage between composition and biological outcomes. This principle is illustrated by studies in which chemically or mechanistically characterized preparations demonstrated reproducible respiratory-protective effects, including thyme extracts with defined antispasmodic and mucociliary activity [104,105], standardized flavonoid-rich preparations in COPD [103], and defined proanthocyanidin preparations in acute lung injury and asthma models [97]. These observations emphasize that mechanistic clarity and phytochemical standardization are essential for meaningful translational interpretation of botanical respiratory therapeutics.

Accordingly, the following sections examine both the mechanistic rationale and the experimental evidence supporting polyphenol-rich herbal mixtures in respiratory diseases. Particular emphasis is placed on phytochemical characterization, adequacy of standardization, and the extent to which reported biological effects can be mapped to established cytoprotective pathways, including antioxidant defense, mitochondrial preservation, inflammasome regulation, barrier protection, macrophage polarization, and anti-fibrotic signaling. This framework is intended to provide a more rigorous and translationally relevant assessment of botanical polyphenols as therapeutic candidates for respiratory medicine.

## 4. Experimental Evidence for Herbal Polyphenolic Mixtures in Respiratory Diseases

### 4.1. Acute Lung Injury and Acute Respiratory Distress Syndrome

Acute lung injury (ALI) and acute respiratory distress syndrome (ARDS) are characterized by diffuse alveolar damage, disruption of epithelial and endothelial barriers, neutrophil infiltration, excessive cytokine release, oxidative stress, pulmonary edema, and severe impairment of gas exchange. Because these syndromes involve rapid amplification of interconnected redox and inflammatory pathways, they represent particularly informative experimental models for evaluating multi-component plant preparations with antioxidant and cytoprotective properties. Experimental studies indicate that polyphenol-rich preparations can reduce pulmonary edema, inflammatory cell recruitment, histopathological injury, oxidative stress markers, and pro-inflammatory cytokine production, while restoring endogenous antioxidant defenses and modulating macrophage polarization, mitochondrial injury, inflammasome activation, and gut–lung axis signaling [37,81,83].

Grape seed proanthocyanidin significantly attenuated LPS-induced acute lung injury by reducing lung pathological injury, inflammatory cytokine expression, and recruitment of monocyte-derived macrophages. Mechanistically, this effect was associated with promotion of macrophage polarization from the M1 phenotype toward the M2a phenotype through activation of the TREM2/PI3K/Akt pathway [97].

Procyanidin B2 further supports the relevance of polymeric phenolics in ALI. In LPS-induced acute lung injury, procyanidin B2 reduced lung edema, histopathological damage, IL-1β, TNF-α, and IL-6 production, while inhibiting M1 macrophage polarization and pyroptosis through suppression of NF-κB/JAK2/STAT1 and NLRP3/caspase-1/ASC signaling [98].

Tea catechin-derived evidence also supports a multi-pathway model of cytoprotection in ALI. EGCG attenuated LPS-induced acute lung injury by reducing pulmonary edema, neutrophil infiltration, myeloperoxidase activity, oxidative damage, and M1 inflammatory mediator expression, while promoting M2 macrophage polarization and KLF4 expression [81]. In acute pancreatitis-associated lung injury, EGCG reduced mitochondrial ROS generation, oxidized mitochondrial DNA accumulation, and NLRP3 inflammasome activation in alveolar macrophages, thereby linking mitochondrial protection to inflammasome suppression [83].

More recently, Rosa roxburghii polyphenols were shown to protect against acute lung injury from the perspective of the lung–gut axis. This study is valuable because it moves beyond a narrow antioxidant model and suggests that the benefits of polyphenol-rich preparations may also involve systemic immunometabolic modulation and microbiota-related mechanisms [37].

Taken together, the ALI/ARDS literature suggests that herbal polyphenolic mixtures are most convincing when they are studied in relation to defined mechanisms such as macrophage polarization, mitochondrial protection, inflammasome regulation, gut–lung axis modulation, oxidative stress suppression, and cytokine control. This makes acute inflammatory lung injury one of the strongest preclinical areas supporting further development of polyphenol-rich respiratory interventions. Representative examples of herbal polyphenolic mixtures and plant-derived extracts investigated in respiratory disease models are summarized in Table 3, with emphasis on phenolic composition, experimental models, mechanistic targets, reported outcomes, and current translational limitations.

### 4.2. Asthma and Allergic Airway Inflammation

Asthma is a heterogeneous airway disease characterized by variable airflow limitation and airway hyperresponsiveness, with contributions from epithelial dysfunction, mucus abnormalities, and structural remodeling. Experimental studies show that allergic asthma is associated with mitochondrial structural abnormalities and epithelial injury, and that severe asthma can exhibit persistent ciliary ultrastructural defects, supporting the view that epithelial dysfunction is a component of disease biology rather than a passive consequence of inflammation [111,112]. In addition, oxidized linoleic-acid metabolites have been shown to promote airway hyperresponsiveness and epithelial injury in experimental systems, while eosinophils can serve as a cellular source of these mediators [113,114,115]. Clinically, asthma also encompasses eosinophilic, neutrophilic, and mixed-granulocytic inflammatory phenotypes, and severe disease may involve steroid-refractory IL-17/Th17-associated pathways [116,117,118,119]. Taken together, the convergence of epithelial injury, redox-active lipid signaling, immune dysregulation, and structural remodeling makes asthma a biologically relevant framework for evaluating multitarget interventions, including polyphenol-rich preparations [120,121].

Several preparations rich in rosmarinic acid and related phenolics have shown beneficial effects in allergic airway models. For example, rosemary-derived preparations and related Lamiaceae phenolics have been associated with attenuation of inflammatory infiltration, oxidative injury, and remodeling-associated changes. This is consistent with broader recent analyses showing that rosmarinic acid-related phytochemicals can modulate immunologic and allergic pathways relevant to airway disease [109].

Rajizadeh et al. reported that inhalation of spray-dried extract of *Salvia rosmarinus* alleviated inflammatory, oxidative, and remodeling changes in asthmatic lungs. This study is particularly valuable because it combines phytochemical relevance with a pulmonary delivery approach, which is directly important for respiratory translation. Rather than treating plant extracts only as oral nutraceuticals, such work begins to address how botanical polyphenols might be formulated for more targeted airway exposure [108].

Likewise, botanical extracts containing rosmarinic acid, caffeic acid derivatives, and related polyphenols have shown reductions in eosinophilic inflammation, tracheal hyperresponsiveness, oxidative stress markers, and pathological changes in experimental allergy models. Although the composition of such extracts is not always reported with ideal depth, the consistency of outcomes across several studies supports the idea that polyphenol-rich mixtures may be especially relevant in airway diseases where inflammation, oxidant stress, and remodeling evolve together [109].

Overall, the asthma literature suggests that herbal polyphenolic mixtures may act through a combined reduction in oxidant burden, inflammatory mediator release, and structural remodeling. However, stronger translational progress will require better standardization of extracts, more direct comparison with standard anti-asthmatic therapy, and more detailed reporting of phytochemical composition.

### 4.3. Pulmonary Fibrosis and Remodeling Disorders

Pulmonary fibrosis represents one of the most challenging respiratory conditions because it reflects not only inflammation and oxidative stress but also persistent dysregulation of wound repair, fibroblast activation, extracellular matrix accumulation, epithelial injury, and irreversible architectural remodeling. Polyphenol-rich plant preparations are particularly attractive in this context because many demonstrate combined antioxidant, anti-inflammatory, and anti-fibrotic activity, which may be more therapeutically relevant than purely anti-inflammatory approaches alone [122].

Experimental studies support the anti-remodeling potential of phenolic-rich herbal preparations in pulmonary fibrosis models. Methanolic extract of Glycyrrhiza glabra attenuated bleomycin-induced pulmonary fibrosis in rats and improved histopathological fibrotic changes, supporting the relevance of herbal preparations in experimental fibrotic lung injury [122]. Additional studies with flavonoid-rich preparations have shown suppression of fibroblast senescence and remodeling-associated signaling; for example, hesperidin alleviated bleomycin-induced pulmonary fibrosis by inhibiting lung fibroblast senescence through the IL-6/STAT3 pathway, accompanied by reductions in p53, p21, p16, α-SMA, and senescence-associated β-galactosidase-positive cells [92].

The broader fibrosis literature also suggests that plant-derived phenolic compounds and mixtures may interfere with profibrotic signaling pathways associated with TGF-β activation, oxidative amplification, epithelial dysfunction, mitochondrial injury, and mesenchymal transition. Curcumin analogue EF24 attenuated experimental pulmonary fibrosis by suppressing alveolar epithelial cell senescence through PTEN activation, inhibition of AKT/mTOR/NF-κB signaling, and restoration of mitophagy [86]. Curcumin also reduced extracellular matrix remodeling and mitochondrial dysfunction in pulmonary fibrosis through regulation of the miR-29a-3p/DNMT3A axis, decreasing collagen I and fibronectin accumulation in lung tissue and pulmonary fibroblasts [91].

Taken together, current evidence suggests that herbal polyphenolic mixtures may be useful in fibrotic lung disease when they combine antioxidant protection, suppression of profibrotic signaling, attenuation of fibroblast activation or senescence, and preservation of epithelial-endothelial integrity. However, the field still requires improved comparative experimental designs, deeper phytochemical characterization, and more convincing translational studies.

### 4.4. Smoke-, Pollutant-, and Noxious Agent-Related Lung Injury

Respiratory injury caused by cigarette smoke, biomass exposure, airborne particulates, mechanical ventilation, and toxic environmental agents represents a major area where oxidative stress and polyphenol pharmacology intersect directly. These exposures generate substantial oxidant burden and trigger secondary inflammatory amplification, mitochondrial dysfunction, epithelial injury, endothelial damage, and remodeling-associated signaling. Consequently, natural product-based interventions, including polyphenol-rich plant extracts, have been investigated as protective strategies in exposure-related and toxin-related lung injury models.

Experimental studies suggest that herbal preparations may attenuate oxidant-induced respiratory injury through combined anti-inflammatory and antioxidant mechanisms. In COPD-related settings, treatment with Zataria multiflora improved inflammatory cytokines, pulmonary function parameters, and respiratory symptoms in patients with COPD, supporting the translational relevance of standardized herbal interventions in chronic inflammatory airway disease [103]. In mechanical ventilation-induced acute lung inflammation, hesperidin reduced inflammatory cell recruitment, oxidative damage, cytokine production, and redox imbalance in vitro and in vivo [93,94].

Collectively, these findings support the concept that plant-derived polyphenolic mixtures may be particularly valuable in exposure-related respiratory diseases because they target multiple interconnected mechanisms simultaneously, including oxidative stress, inflammatory amplification, epithelial dysfunction, and remodeling-associated signaling. Nevertheless, substantial heterogeneity in extract composition, experimental design, and mechanistic characterization still limits direct translational interpretation, emphasizing the need for better standardized and clinically relevant respiratory studies.

### 4.5. Overall Interpretation of the Preclinical Evidence

The preclinical literature on herbal polyphenolic mixtures in respiratory disease is encouraging but uneven in quality. The strongest studies are those that include at least four elements: first, a reasonably characterized extract or fraction; second, a well-defined disease model; third, mechanistic data linking the preparation to known redox or inflammatory pathways; and fourth, concordance between biochemical, histological, and functional outcomes. Studies lacking these features may still be of interest, but they are less suitable as core evidence in a high-quality review [7,123].

Overall, the most convincing mechanistic pattern across preclinical studies is the convergence on a limited number of recurring targets: NOX-derived ROS production, Nrf2/HO-1 activation, NF-κB suppression, mitochondrial protection, inflammasome attenuation, and control of fibrotic remodeling. This recurring pattern strongly supports the view that polyphenol-rich herbal mixtures are not acting through random nonspecific effects but through biologically meaningful cytoprotective networks relevant to respiratory disease.

## 5. Translational Challenges

One of the major barriers to the clinical translation of polyphenol-based respiratory interventions is the discrepancy between strong preclinical activity and inconsistent human applicability. Many polyphenols reduce oxidative stress, inflammation, and tissue injury in cell and animal models; however, their effective concentrations in vitro often exceed levels achievable in human plasma or lung tissue after conventional oral administration. This limitation is largely explained by poor aqueous solubility, chemical instability, limited intestinal absorption, extensive phase II metabolism, microbial biotransformation, and rapid systemic clearance [124,125,126]. This issue is even more complex for herbal mixtures, where different constituents may have distinct absorption profiles, metabolites, tissue distribution patterns, and half-lives.

A second major challenge is that the biological activity of polyphenols is often mediated not by the parent compound, but by circulating conjugates or gut microbiota-derived metabolites. After ingestion, many flavonoids and phenolic acids undergo glucuronidation, sulfation, methylation, and microbial ring fission, generating metabolites with pharmacological properties that may differ substantially from those of the original compound [127]. Therefore, mechanistic studies using unmetabolized parent compounds at high concentrations may not accurately reflect the situation in vivo. Future respiratory studies should increasingly test physiologically relevant metabolites and concentrations rather than relying exclusively on parent molecules.

Variability in botanical composition represents another critical translational barrier. Herbal polyphenolic mixtures are often described broadly as “polyphenol-rich extracts”, but preparations may differ markedly in plant species, cultivar, geographic origin, harvest conditions, plant part, extraction solvent, processing method, storage conditions, and concentration of marker compounds. As a result, two extracts derived from the same botanical species may produce different biological effects if their phenolic profiles are not comparable. For this reason, high-quality studies should include botanical authentication, quantitative phytochemical profiling, batch-to-batch consistency testing, and clear reporting of marker compounds using validated analytical methods such as HPLC, LC-MS/MS, or HPTLC [128,129,130].

Route of administration is particularly important in respiratory applications. Although oral delivery is convenient and widely used, it may be inefficient for targeting lung tissue because of first-pass metabolism and variable systemic exposure. Pulmonary delivery offers a more direct strategy by increasing local airway or alveolar exposure while potentially reducing systemic adverse effects. This is supported by experimental work showing that inhaled spray-dried *Salvia rosmarinus* extract reduced inflammatory, oxidative, and remodeling changes in asthmatic rat lungs [108]. However, pulmonary delivery also introduces formulation challenges, including particle size control, aerosol performance, lung deposition, mucociliary clearance, macrophage uptake, stability, and local tolerability.

Formulation science is therefore central to future translation. Nanoformulations, liposomes, polymeric nanoparticles, solid lipid nanoparticles, phytosomes, cyclodextrin complexes, and dry powder inhalation systems may improve solubility, stability, pulmonary deposition, and controlled release of polyphenols. Recent reviews of inhaled nanoparticle-based therapy emphasize that pulmonary nanocarriers can improve residence time and local drug concentration, but their safety, scalability, reproducibility, and long-term pulmonary biocompatibility must be carefully evaluated before clinical use [131,132]. For herbal mixtures, formulation is even more challenging because multiple active constituents may differ in polarity, stability, degradation profile, and release kinetics.

Clinical evidence remains the weakest part of the field. Most studies on herbal polyphenolic mixtures in respiratory diseases are preclinical, while human trials are limited, heterogeneous, and often insufficiently standardized. Differences in extract composition, dose, treatment duration, route of administration, patient phenotype, disease severity, background therapy, and outcome measures make it difficult to compare results or draw firm conclusions. Future clinical studies should use standardized extracts, predefined marker compounds, appropriate comparators, and clinically meaningful endpoints such as lung function, exacerbation frequency, symptom scores, imaging outcomes, inflammatory biomarkers, oxidative stress markers, and safety parameters. For chronic diseases such as asthma, COPD, and pulmonary fibrosis, long-term follow-up will also be necessary to determine whether molecular and biomarker improvements translate into durable clinical benefit.

Beyond the limited number of clinical studies, an important weakness of the current evidence base is the substantial heterogeneity of experimental designs. Studies differ markedly in botanical source material, extraction procedures, phytochemical characterization, dosing regimens, treatment duration, disease models, and outcome measures. As a result, direct comparison between studies is often difficult, even when similar respiratory indications are investigated. Moreover, many experimental reports focus primarily on demonstrating biological activity, while comparatively fewer studies provide comprehensive characterization of the tested preparations or establish clear exposure–response relationships. This heterogeneity limits reproducibility and complicates the translation of promising preclinical findings into clinically applicable interventions.

Another important limitation is the frequent overinterpretation of antioxidant activity. Many botanical studies still rely heavily on descriptive assays or broad claims of “antioxidant” and “anti-inflammatory” effects without sufficient mechanistic validation. To move beyond this stage, future studies should link chemical composition to defined molecular targets, including Nrf2/HO-1 activation, NF-κB and MAPK suppression, NOX-dependent ROS inhibition, mitochondrial preservation, inflammasome modulation, epithelial–endothelial barrier protection, and TGF-β-associated anti-fibrotic signaling. This mechanism-driven approach would make it possible to distinguish genuinely promising preparations from extracts that show only nonspecific activity under experimental conditions.

An additional concern is that many studies continue to evaluate respiratory protection using relatively nonspecific biomarkers of oxidative stress and inflammation without adequately addressing disease-specific mechanisms. While reductions in reactive oxygen species, lipid peroxidation, or pro-inflammatory cytokines are frequently reported, these endpoints alone may not fully explain improvements in complex respiratory disorders such as pulmonary fibrosis, asthma, COPD, or pulmonary hypertension. Future investigations should therefore prioritize mechanistic validation using pathway-specific biomarkers, target engagement studies, and integrated multi-omics approaches to better define how polyphenolic preparations influence disease progression.

From our perspective, the current body of evidence suggests that the strongest support for polyphenolic interventions exists in acute inflammatory and oxidative lung injury models, where protective effects have been reproduced across multiple experimental systems and are supported by relatively consistent mechanistic findings involving redox regulation, inflammatory signaling, and preservation of tissue integrity. Evidence for anti-fibrotic, anti-remodeling, and pulmonary vascular protective effects is also encouraging, but remains comparatively less mature and is frequently based on a limited number of studies, making independent validation an important priority.

An additional unresolved question is whether complex polyphenolic mixtures consistently provide advantages over individual purified compounds. Although multi-component preparations may theoretically offer broader biological coverage through simultaneous modulation of multiple pathways, direct comparative studies remain scarce. Consequently, it is still difficult to determine whether the observed benefits arise from genuine synergistic interactions, additive effects, or simply the activity of dominant constituents within the mixture. Addressing this question will be important for the rational development of future botanical therapeutics.

In our opinion, future progress in this field will depend less on the discovery of additional polyphenolic preparations and more on improving study quality and translational relevance. Priority should be given to rigorous phytochemical standardization, pharmacokinetic characterization, validation of target engagement, clinically relevant dosing strategies, and the development of lung-targeted delivery systems. Equally important will be the identification of robust translational biomarkers capable of linking molecular responses observed in experimental models with clinically meaningful outcomes in patients. Such advances will be essential for determining whether herbal polyphenolic mixtures can ultimately evolve from promising experimental interventions into evidence-based therapeutic options for respiratory diseases.

## 6. Materials and Methods

A structured literature search was conducted to identify relevant studies on oxidative stress, polyphenols, and respiratory diseases. The primary databases used were PubMed, Scopus, and Web of Science.

The search covered publications from approximately 2005 to 2025, with particular emphasis on studies published within the last 10 years. The following keywords and combinations were used:

“polyphenols”, “herbal extracts”, “oxidative stress”, “reactive oxygen species”, “respiratory diseases”, “COPD”, “asthma”, “acute lung injury”, “pulmonary fibrosis”, “Nrf2”, “NF-κB”, “inflammasome”, and “mitochondrial dysfunction”.

Boolean operators (AND/OR) were applied to refine the search strategy.

Inclusion criteria:

- Experimental (in vitro and in vivo) studies investigating polyphenols or polyphenol-rich plant extracts in respiratory models
- Mechanistic studies addressing oxidative stress, inflammation, and cytoprotective pathways
- Reviews providing relevant background on molecular pathways
- Articles published in English

Exclusion criteria:

- Studies lacking mechanistic relevance to oxidative stress or cytoprotection
- Reports with insufficient methodological detail
- Retracted or unreliable publications

Priority was given to well-characterized experimental studies, particularly those linking polyphenol activity to defined molecular pathways such as Nrf2/HO-1, NF-κB, MAPK, mitochondrial regulation, and inflammasome signaling.

The final selection of references was based on scientific relevance, mechanistic depth, and translational importance, rather than meta-analytic quantitative synthesis.

## 7. Conclusions

Herbal polyphenolic mixtures represent promising multi-target modulators of oxidative stress, inflammation, and tissue remodeling in respiratory diseases. Preclinical evidence consistently shows that these preparations can influence key pathogenic pathways, including activation of the Nrf2/HO-1 antioxidant system, suppression of NF-κB and MAPK signaling, regulation of mitochondrial function, and attenuation of profibrotic processes.

The principal message emerging from the current evidence is that herbal polyphenolic mixtures should not be viewed merely as sources of antioxidant molecules. Rather, they represent complex multi-component systems capable of simultaneously modulating oxidative stress, inflammatory signaling, mitochondrial dysfunction, epithelial–endothelial barrier injury, and tissue remodeling. This systems-level activity distinguishes them conceptually from isolated antioxidant interventions.

Nevertheless, substantial challenges remain before these preparations can be incorporated into evidence-based respiratory medicine. Standardization of botanical preparations, rigorous phytochemical characterization, optimization of bioavailability, and high-quality randomized clinical trials are essential prerequisites for successful translation. Future research should focus not only on demonstrating efficacy but also on establishing reproducible and clinically relevant formulations suitable for regulatory evaluation and therapeutic implementation.

## Figures and Tables

**Table 1:** Major molecular markers of oxidative stress and cytoprotection in respiratory diseases.

Pathway/Marker	Main Function	Dysregulation in Respiratory Disease	Relevance for Polyphenol-Based Cytoprotection	References
Nrf2	Master transcription factor regulating antioxidant and phase II defense genes	Often functionally impaired or insufficiently activated under chronic oxidant stress; associated with reduced cellular adaptation in COPD, asthma, and inflammatory lung injury	Many polyphenols activate Nrf2 signaling and restore endogenous antioxidant capacity	[2,6]
HO-1	Stress-inducible antioxidant and cytoprotective enzyme downstream of Nrf2	Reduced induction or insufficient activity may enhance oxidative injury and inflammation	Frequently upregulated by polyphenols; contributes to anti-inflammatory and anti-apoptotic responses	[6,52]
SOD	Converts superoxide anion into hydrogen peroxide	Activity may be reduced in chronic oxidative lung disorders	Polyphenols can enhance antioxidant enzyme status and reduce ROS accumulation	[1,53]
CAT	Converts hydrogen peroxide into water and oxygen	Reduced activity contributes to oxidative amplification	Restoration of CAT activity is part of the antioxidant effect of several polyphenols	[53,54]
GPx	Detoxifies hydrogen peroxide and lipid hydroperoxides using glutathione	Impaired function increases membrane lipid oxidation and cellular injury	Polyphenols may support GPx-linked redox protection directly or through Nrf2-dependent pathways	[1,2]
GSH	Major intracellular non-enzymatic antioxidant; maintains thiol redox balance	Depletion increases susceptibility to epithelial and endothelial injury	Polyphenols may preserve glutathione status and reinforce glutathione-dependent defense systems	[3,5]
NF-κB	Central inflammatory transcription factor	Overactivated in chronic airway inflammation, smoke-induced injury, and acute inflammatory lung damage	Polyphenols often suppress NF-κB activation, reducing cytokine and mediator expression	[4,7]
MAPK (p38, ERK, JNK)	Stress-responsive kinase pathways regulating inflammation, apoptosis, remodeling, and mucus production	Frequently overactivated in oxidative and inflammatory respiratory conditions	Polyphenols may attenuate MAPK activation and reduce downstream tissue injury	[1,49]
NLRP3 inflammasome	Activates caspase-1 and promotes IL-1β/IL-18 maturation and pyroptosis	Contributes to smoke-induced inflammation, epithelial injury, and acute inflammatory responses	Several polyphenols inhibit inflammasome activation and downstream inflammatory damage	[48,55]
NOX2/NADPH oxidase	Major enzymatic source of ROS in inflammatory cells and injured lung tissue	Upregulated during inflammation, infection, and smoke exposure; amplifies oxidative injury	Some plant polyphenols suppress NOX-dependent ROS generation	[1,52]
Mitochondrial ROS/DJ-1-related mitochondrial protection	Controls energy metabolism, redox buffering, and cell survival	Mitochondrial dysfunction promotes ROS overproduction, apoptosis, and barrier failure	Polyphenols may preserve mitochondrial integrity and reduce oxidant-driven injury	[1,8]
Endothelial-to-mesenchymal transition (EndMT)	Phenotypic transition contributing to vascular remodeling and fibrosis	Promotes endothelial dysfunction and chronic remodeling in pulmonary vascular disease	Polyphenols may inhibit EndMT and preserve endothelial phenotype	[56]
TGF-β-associated profibrotic signaling	Drives fibroblast activation, extracellular matrix deposition, and fibrotic remodeling	Persistently activated in pulmonary fibrosis and chronic remodeling states	Several polyphenols show anti-fibrotic and anti-remodeling effects	[9,57]

**Table 2:** Major individual polyphenols relevant to respiratory diseases.

Polyphenol	Chemical Class	Major Natural Sources	Main Respiratory-Relevant Mechanisms	Respiratory Conditions/Models Discussed in the Literature	Main Limitations	References
Quercetin	Flavonol	Onion, apple, berries, many medicinal plants	Antioxidant activity; suppression of NF-κB; activation of Nrf2-related defense; inhibition of NLRP3 inflammasome; attenuation of ferroptosis/pyroptosis-related injury	Acute lung injury, inflammatory airway injury, pulmonary fibrosis	Low oral bioavailability; formulation dependence; mostly preclinical evidence	[55,69,70,71]
Resveratrol	Stilbene	Grapes, berries, peanuts	Anti-inflammatory activity; suppression of oxidative stress; interference with TGF-β-associated profibrotic signaling; anti-remodeling effects	Airway inflammation, smoke-related injury, pulmonary fibrosis	Poor bioavailability; limited clinical validation in respiratory disease	[72,73,74,75,76]
Rosmarinic acid	Phenolic acid derivative	Rosemary, perilla, basil, lemon balm	Antioxidant action; reduction in airway inflammation; modulation of oxidative enzymes; possible anti-remodeling effects	Allergic asthma, eosinophilic airway inflammation	Mainly preclinical evidence	[54,77,78,79,80]
EGCG	Flavan-3-ol (catechin)	Green tea	Antioxidant, anti-inflammatory, anti-fibrotic, apoptosis-modulating effects	Asthma, ALI, fibrotic lung disorders	Stability and bioavailability issues	[81,82,83,84,85]
Curcumin	Polyphenolic diarylheptanoid	Turmeric	Antioxidant and anti-inflammatory effects; cytokine modulation	COPD, inflammatory lung injury	Very poor bioavailability	[86,87,88,89,90,91]
Hesperidin	Flavanone glycoside	Citrus fruits	Antioxidant activity; anti-inflammatory effects	Inflammatory lung disease, COPD models	Limited clinical data	[92,93,94,95,96]
Tannins	Polymeric phenolics	Various plants	Antioxidant, anti-inflammatory, barrier-modulating effects	Non-malignant respiratory disorders	Heterogeneous composition	[97,98,99,100,101]

**Table 3:** Representative herbal polyphenolic mixtures and extracts investigated in respiratory disease models.

Herbal Preparation/Plant Source	Major Reported Phenolic Constituents Or Profile	Respiratory Model	Route/Regimen	Main Mechanistic Targets	Main Outcomes	Main Limitations	Reference
*Securidaca inappendiculata* polyphenols	Polyphenol-rich fraction; exact composition partly characterized	Acute lung injury in rats	Experimental systemic administration in rat ALI model	Oxidative stress-sensitive pathways; inflammatory suppression	Reduced lung injury, improved oxidative stress profile, anti-inflammatory effects	Limited external validation; extract complexity	[106]
*Bletilla striata* phenolic-rich fraction	Phenolic-enriched active fraction	LPS-induced acute lung injury	Experimental administration in murine/rodent ALI model	p47phox/NOX2 inhibition; Nrf2/HO-1 activation	Reduced oxidative injury and inflammation; improved histology	Need for broader replication and standardization	[52]
*Nymphaea candida* total polyphenols	Total polyphenol fraction	Septic/LPS-induced acute lung injury	Preventive administration in experimental model	Anti-inflammatory and antioxidant actions	Protection against septic ALI-associated tissue injury	Limited translational depth; composition requires fuller standardization	[63]
*Rosa roxburghii* polyphenols	Polyphenol-rich fruit-derived preparation	Acute lung injury	Experimental administration	Oxidative stress control; inflammation modulation; possible microbiota/lung-gut axis effects	Reduced ALI severity with broader systemic mechanistic interpretation	Novel mechanism attractive but requires validation	[37]
Rosemary leaf extract (*Rosmarinus officinalis/Salvia rosmarinus*)	Rosmarinic acid, carnosic acid, carnosol, related phenolics	Bleomycin-induced pulmonary fibrosis	Repeated administration after fibrosis induction in rats	Antioxidant and anti-fibrotic actions	Improvement of oxidative stress parameters and attenuation of fibrotic changes	Extract variability; no human validation	[107]
Spray-dried extract of *Salvia rosmarinus*	Rosmarinic acid-related phenolics and rosemary phytochemicals	Experimental asthma	Inhalation delivery	Anti-inflammatory, antioxidant, anti-remodeling	Reduced asthmatic inflammatory, oxidative, and remodeling changes	Requires further pharmacokinetic and comparative studies	[108]
Rosmarinic acid-rich Lamiaceae preparations	Rosmarinic acid with associated phenolic compounds	Allergic airway inflammation/asthma	Variable across studies	Oxidative enzyme modulation; inflammatory suppression	Lower eosinophilic inflammation, reduced airway hyperresponsiveness, improved oxidative indices	Heterogeneous preparations and protocols	[109]
Ziziphus spina-christi extract	Polyphenol-containing botanical extract	Bleomycin-induced fibrosis	Experimental administration	Antioxidant and anti-fibrotic pathways	Attenuation of fibrosis-associated injury	Early-stage evidence	[110]

## Data Availability

No new data were created or analyzed in this study. Data sharing is not applicable to this article.
